# Human Coaching Methodologies for Automatic Electronic Coaching (eCoaching) as Behavioral Interventions With Information and Communication Technology: Systematic Review

**DOI:** 10.2196/23533

**Published:** 2021-03-24

**Authors:** Ayan Chatterjee, Martin Gerdes, Andreas Prinz, Santiago Martinez

**Affiliations:** 1 Department for Information and Communication Technologies Centre for e-Health University of Agder Grimstad Norway; 2 Department of Health and Nursing Science Centre for e-Health University of Agder Grimstad Norway

**Keywords:** coaching, electronic coaching, human behavior, healthy lifestyle, persuasive technology

## Abstract

**Background:**

We systematically reviewed the literature on human coaching to identify different coaching *processes* as behavioral interventions and *methods* within those processes. We then reviewed how those identified coaching processes and the used methods can be utilized to improve an electronic coaching (eCoaching) process for the promotion of a healthy lifestyle with the support of information and communication technology (ICT).

**Objective:**

This study aimed to identify coaching and eCoaching processes as behavioral interventions and the methods behind these processes. Here, we mainly looked at processes (and corresponding models that describe coaching as certain processes) and the methods that were used within the different processes. Several methods will be part of multiple processes. Certain processes (or the corresponding models) will be applicable for both human coaching and eCoaching.

**Methods:**

We performed a systematic literature review to search the scientific databases EBSCOhost, Scopus, ACM, Nature, SpringerLink, IEEE Xplore, MDPI, Google Scholar, and PubMed for publications that included personal coaching (from 2000 to 2019) and persuasive eCoaching as behavioral interventions for a healthy lifestyle (from 2014 to 2019). The PRISMA (Preferred Reporting Items for Systematic Reviews and Meta-Analyses) framework was used for the evidence-based systematic review and meta-analysis.

**Results:**

The systematic search resulted in 79 publications, including 72 papers and seven books. Of these, 53 were related to behavioral interventions by eCoaching and the remaining 26 were related to human coaching. The most utilized persuasive eCoaching methods were personalization (n=19), interaction and cocreation (n=17), technology adoption for behavior change (n= 17), goal setting and evaluation (n=16), persuasion (n=15), automation (n=14), and lifestyle change (n=14). The most relevant methods for human coaching were behavior (n=23), methodology (n=10), psychology (n=9), and mentoring (n=6). Here, “n” signifies the total number of articles where the respective method was identified. In this study, we focused on different coaching methods to understand the psychology, behavioral science, coaching philosophy, and essential coaching processes for effective coaching. We have discussed how we can integrate the obtained knowledge into the eCoaching process for healthy lifestyle management using ICT. We identified that knowledge, coaching skills, observation, interaction, ethics, trust, efficacy study, coaching experience, pragmatism, intervention, goal setting, and evaluation of coaching processes are relevant for eCoaching.

**Conclusions:**

This systematic literature review selected processes, associated methods, strengths, and limitations for behavioral interventions from established coaching models. The identified methods of coaching point toward integrating human psychology in eCoaching to develop effective intervention plans for healthy lifestyle management and overcome the existing limitations of human coaching.

## Introduction

### Overview

A coach [1-4] is a trusted role model, adviser, wise person, friend, mensch (a person of integrity and honor), steward (supervisor), or guide. A coach facilitates experimental learning that results in future-oriented abilities. Coaches can shape new visions and plans to achieve desired results. Coaching has been implemented in management, leadership, entrepreneurship, health care, and performance management. It helps participants to cultivate themselves and become more successful in achieving their set goals. Successful coaching relies on a good relationship, mutual trust, and freedom of expression between coaches and participants [1-6]. Effective coaching leads to excellent performance, self-motivation, and self-correction. Coaching processes can be divided into the following two categories: (1) traditional offline human coaching (coaching by humans) and (2) electronic coaching (eCoaching).

Traditional offline human coaching processes involve the following methods [1,4,7-10]: privacy, focus, goal orientation, performance improvement, and trust. The process associated with coaching by humans can be achieved either face-to-face or remotely (via telematic means). Furthermore, the coaching process can be categorized [5-16] as health coaching to address negative behavioral change, cognitive-behavioral coaching, mental health coaching, in-house executive coaching in businesses, companies, or industries (corporate coaching), sports coaching, motivational coaching, educational coaching, and coaching to carry out activities of daily living. Traditional human coaching is a dialogic, goal-oriented, pragmatic learning practice. The human coaching process can be further enhanced through electronic modes, such as video, audio, email, chatbot, and text, with the support of information and communication technology (ICT), which is referred to as eCoaching. In the last decade, personal coaching for behavioral intervention has been increasingly used to promote a healthy lifestyle [17,18]. eHealth uses ICT for health [19,20]. eCoaching is a promising eHealth research direction for continuous customized ways of lifestyle support [21,22]. It is an evolution of offline human coaching with the flexibility of electronic services allowing ubiquitous access to the process. eCoaching technologies represent an evolving trend in the domain of human behavioral intervention. The coaching core behind an eCoaching system can be a human (eg, telemedicine), an artificial intelligence (AI) agent (eg, algorithm), or a combination of these. An eCoaching system consists of a set of programmed modules representing an artificial entity that may look at, query, examine from, and predict a consumer’s behaviors in a specific context and in a specific period. Application domains of eCoaching include the following [18,23-55]: nutrition coaching, physical activity coaching, coaching for mental health, coaching for activities of daily living in the elderly, diabetic coaching, and cardiac rehabilitation. Studies in eCoaching can offer methods to enhance individual healthcare with ICT. A virtual eCoaching recommendation system can guide people and convey the appropriate recommendations in real time to improve their lifestyle [21,22,56]. The leading methods of eCoaching processes are monitoring, decision making, goal setting, persuasion, awareness provision (intervention), goal evaluation, and learning for future actions [24,27,32-34,57-59]. Digital techniques of lifestyle change with eCoaching have appeared as efficient and scalable options for intensive behavioral counseling when face-to-face or in-person programs are inaccessible or undesirable. eCoaching can make human behavioral interventions useful when combined with human coaching methods [57,60,61]. In the eCoaching processes, participants can remotely take part and avoid traveling, expenses, and transport risks. It is relevant to note that eCoaching will electronically handle data. Therefore, complying with general data protection regulations is critical for the safety and security of participants. eCoaching processes may ideally influence health outcomes, for which aspects, such as usability, efficacy, and adherence, may play important roles to influence health and/or health behavior. “Efficacy” means the effects of behavioral intervention following any coaching process (of any method, not only of eCoaching). “Usability” means the effectiveness, efficiency, and satisfaction when using a technology. “Adherence” means the degree to which the technology is used as intended [7,57,58].

Coaching as a human behavioral intervention is a personalized planned process designed to reward and reinforce the positive behavior of human beings. Each behavioral intervention differs from others based on the participants who are the primary targets of the intervention, where psychology and context play crucial roles [21,56,60]. The methods of a successful behavioral intervention plan “focus” on the identification of problems, the analysis of identified problems, prevention strategies and modification techniques, encouragement or motivation, strategic planning to diminish negative behavior, and participant engagement [1-3,5,35,56,60,62]. The coaching process for behavioral intervention should include appropriate guidelines, mutual trust, a rewarding plan, participant feedback, goal setting, and goal evaluation methods to make it useful for coaching and eCoaching (coaching by an electronic coach [eCoach]) processes [1,23,63]. Time is a critical factor in determining the format of coaching. Integration of coaching methodologies into persuasive eCoaching for electronic personalized behavioral interventions creates new opportunities for a healthy lifestyle [1-3]. It is rewarding for participants to change negative behavior using evidence-based methods and to observe the increase in their health and strength [4,5,60].

### Aim of the Study

The aim of this systematic literature review was to identify key processes from the coaching methodologies to tackle the existing challenges coupled with the human coaching and eCoaching processes as behavioral interventions. The focus on coaching is justified by the fact that health and wellness remote coaches are an asset to clinical practice although they are underutilized in the health care system [6,21,56].

This systematic literature review addresses the following research questions (RQs):

(1) RQ1: What are the existing human coaching processes?

(2) RQ2: Which conceptual coaching models can be used to explain the coaching process?

(3) RQ3: What are the basic coaching methods to make coaching processes successful for the promotion of a behavioral intervention?

(4) RQ4: How can the methods of human coaching processes be incorporated into eCoaching for behavioral intervention to promote a healthy lifestyle?

(5) RQ5: How can eCoaching promote a healthy lifestyle with proven coaching methods using ICT?

## Methods

A systematic literature review was used to acquire a comprehensive overview of the current literature on the topic in a reproducible and transparent way. Systematic reviews represent a scientific synthesis [64,65] of evidence. The PRISMA (Preferred Reporting Items for Systematic Reviews and Meta-Analyses) evidence-based framework [64] was used for the systematic review and meta-analyses. Initially, we performed a random search in the “Google Scholar” database with the following four key terms: “coaching,” “electronic coaching,” “eCoaching,” and “e-Coaching” (see Table 1 for the results of the initial random search). It was observed that the keyword “electronic coaching” obtained the greatest number of results among the last three key terms.

**Table 1:** Initial “Google Scholar” random literature search results according to publication year.

Key terms	1998-2019	2008-2019	2014-2019	2017-2019
Coaching, n	482,000	497,000	159,000	50,800
Electronic coaching, n	133,000	72,600	25,700	18,400
eCoaching, n	396	347	270	184
e-Coaching, n	5740	5100	3300	1710

Subsequently, literature searches were conducted with selected search string patterns (Table 2) on the following electronic databases, as they compiled the greatest number of scientific sources related to coaching and eCoaching studies: Google Scholar, EBSCOhost, Scopus, ACM, Nature, SpringerLink, IEEE Xplore, MDPI, and PubMed. This study’s search strategy was created in collaboration with the library of the University of Agder (UiA) in Norway, based on the following two main search topics: (1) coaching as a behavioral intervention and (2) eCoaching as a behavioral intervention. Related search keywords were identified using MeSH (Medical Subject Headings) terms, synonyms, keywords from relevant articles, and self-determined search terms. The means, such as EndNote (V. X9), DOAJ, Sherpa/Romeo, and Microsoft Excel (MS Office 365 V. 16.x), were used to effectively search, collect, and select related articles. We aimed to include articles that described coaching methodologies and eCoaching related to behavioral interventions. Articles were categorized among the groups quantitative, qualitative, and editorial. The quantitative study deals with statistical analysis on systematically collected data to test a specific hypothesis, while the qualitative study focuses on words and meanings to explore ideas and experiences in depth. The search results are depicted in Multimedia Appendix 1. We included articles based on the following inclusion criteria: (1) peer-reviewed, full length articles written in English, (2) eCoach articles published in the selected databases between 2014 and 2019, (3) coaching articles published in the selected databases between 2000 and 2019, (4) articles indexed in “Google Scholar,” (5) journal papers, conference papers, or books, (6) qualitative (primary and secondary research) and quantitative studies, and (7) coaching articles related to human behavioral intervention. The traditional offline human coaching processes are older than eCoaching processes. Thus, the period for searching the selected electronic databases differs for “coaching” and “eCoaching.”

**Table 2:** Search strings used for article searching.

Category	Search strings	Publication year
eCoach	(mentoring OR “e-coach” OR “ecoach” OR “electronic coach*” OR counseling OR educat* OR electro*coach*) OR (telemedic* OR “mobile health” OR mhealth OR ehealth OR “e-therap*” OR “e-counseling”) AND (obesity OR overweight OR overnutrit* OR hypernutrit* OR lifestyle OR behavior OR behaviour) AND (persuasion OR recommendation OR intervention)	2014-2019
Coach	(mentoring OR coaching OR counseling OR educat* OR coach* OR executive* OR sport* OR activity* OR life*) AND (health* OR behavior OR behaviour OR psychology OR lifestyle)	2000-2019

**Figure 1:**
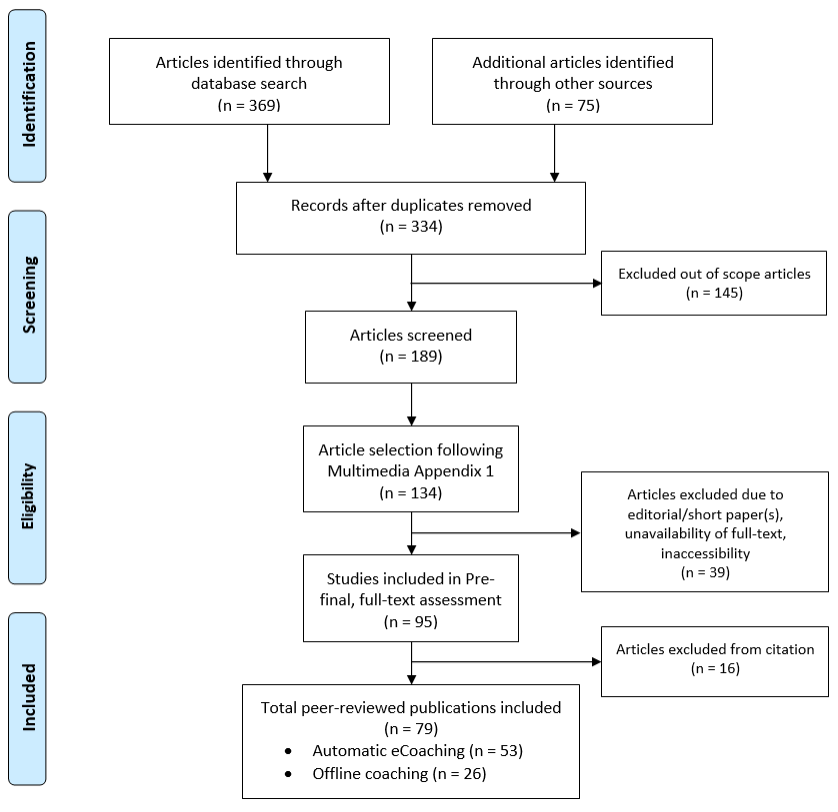
PRISMA (Preferred Reporting Items for Systematic Reviews and Meta-Analyses) flowchart for the article selection process.

We excluded editorial articles, studies related to robotic coaching, philosophical papers, articles with a lot of similar content or articles that were exactly repeated, and articles that were neither “open access” nor accessible through the university library. The full process of selecting sources for this review is depicted in a flowchart (Figure 1). The process includes the following four phases [64]: identification, screening, eligibility, and inclusion.

## Results

### Literature Search Results

The searches (electronic database and manual searches) resulted in 444 papers (369 in electronic databases and 75 identified manually), where 110 were duplicates. In the prefinal stage, we selected 95 articles for full-text review after checking the abstract, conclusion, length of the paper, and availability of full text. In the final search, we included peer-reviewed publications only, resulting in 79 core peer-reviewed articles related to “coaching” (53 papers) and “eCoaching” (26 papers). The categorical distribution (quantitative/qualitative) of the selected articles under “eCoaching” and “coaching” was as follows: coaching (23 quantitative, 30 qualitative) and eCoaching (7 quantitative, 19 qualitative).

This systematic literature review identified different coaching process descriptive models, as well as how they are carried out and in which context. We observed underlying theories to support traditional human coaching processes, such as hope theory [7] and amoeba theory [2], and different terms associated with both coaching and eCoaching processes, such as components, conceptual models, aspects, principles, concepts, activities, and methods. The usage of heterogenous terms to describe similar or nearly similar coaching and eCoaching processes resulted in ambiguity, less contentedness, and reduced clarity. Therefore, throughout the study, we concentrated on *processes* and *methods* to answer and discuss our research questions. They can be explained as follows: *processes* describe different coaching and eCoaching models and their implementation style, and the success of a coaching and eCoaching process depends on the adopted *methods*. Their evaluation helps to determine the performance of the human coaching and eCoaching processes.

The identified methods help us to understand the principles, strategies, effectiveness, and constraints of coaching and eCoaching processes. An eCoach may create optimized, real-time, comprehensible, automated, contextual, evidence-based, and personalized intervention strategies for participants. Moreover, an eCoach may address the challenges associated with coaching, such as scope, the volume of the target audience, bias, cost, automation, accessibility, security, flexibility, credibility, conceptual clarity, location, and time independence, as revealed from the systematic literature review [2,4,6,21,35,56,62,66-70]. This systematic literature review identified 21 studies contributing to answering RQ1 regarding coaching methodologies; 17 studies contributing to answering RQ2 regarding a conceptual coaching model; 20 studies contributing to answering RQ3 regarding coaching methods for the promotion of “behavioral intervention;” 59 studies contributing to answering RQ4 regarding the integration of “coaching process” into “eCoaching for behavioral intervention;” and 35 studies contributing to answering RQ5 to advance “eCoaching for behavioral intervention” for a “healthy lifestyle” with proven “coaching methodologies” using ICT (several included overlapped studies contribute to multiple RQs).

### RQ1: What Are the Existing Human Coaching Processes?

Bartlett [1] proposed a method where mutual trust, respect, and freedom of expression were considered as the elements of a successful coaching relationship. The model combines the establishment of a relationship between a coach and a trainee, recognizing an opening to assess obstacles related to coaching, observation, and assessment; enrollment of clients; and coaching conversations. Potrac et al [66] proposed another model that combines systematic observation and interpretive interview techniques to gain a deeper and broader understanding of personal coaching’s instructional process. The suggested multimethod framework concerns identification of the instructional behaviors within the practice environment, generation of the understanding of why coaches behave as they do within the practice environment, and examination of the impact on the instructional strategies and understanding by humans. Cunningham et al [9] recommended a model based on hierarchical regression analysis with a stepwise process to show that an earlier success accuracy, collective coaching experience, collective professional coaching experience, and racial diversity are significantly associated with team performance. Côté [5] proposed that informal self-directed learning modes have relatively more significance than formal and nonformal learning. The proposed model combines the following three variables: (1) individuals with different backgrounds, experiences, and knowledge, (2) coaching work in various types of contexts with varying amounts of resources, equipment, and facilities, and (3) coaching work with participants varying in terms of age, developmental level, and goals. Their proposed coaching model divides variables into the two categories of ambient components (such as coach’s and participant’s characteristics, and contextual factors) and behavioral components (such as competition, organization, and training). The model proposed by Green et al [7] included the concept of coaching psychology and hope theory, based on the belief that human actions are goal directed. They claimed that the cognitive-behavioral solution-focused coaching model provides preliminary evidence on life coaching that can enhance mental health, quality of life, and goal attainment. Goal setting and goal evaluation are central to lifestyle coaching and are the pillars of successful self-regulation. The coaching study focused on evaluating the effectiveness of a cognitive-behavioral, solution-focused, life coaching group program, and its impact on goal striving, well-being, and hope. The assessment included measures of the “Satisfaction with Life Scale (SWLS)” and the “Positive and Negative Affect Scale (PANAS).” Murphy et al [6] proposed a model focused on executive coaching. With the support of conceptual clarity, executive coaching could unify efforts and resources and provide a common understanding to enhance human resource developmental programs. The human resource should play an active role in developing the organizational capacity for leadership. The proposed model of Richards [67] combines a recurrent process of suitable environment creation for coaching, learning for innovation and successful adaptation, and achievement (coaching performance) for sustained performance. Coaches need to rethink the discipline of coaching as if it is performed well, and coaching can increase motivation and contribute to sustaining high performance. Another model proposed by Richards [67] combines “tell” and “do” instructions. The model is based on the method of conventional thinking to improve a participant’s performance by telling them or showing them what they are doing wrong in order to avoid any repetitive mistakes. This model is beneficial within a short-term context and frame of mind. However, overuse of the approach will undermine efforts to achieve long-term performance. The proposed model by Flaherty [2] was drawn from the concept of phenomenology and combines the following five methods in the coaching process: relationship building (based on mutual satisfaction, mutual respect, mutual trust, and freedom of expression), pragmatism (persistent correction following a feedback loop), two tracks (client and coach engagement), always/already (intervention planning), and identification of techniques that do not work (identification of challenges/ limitations). The proposed amoeba theory is discussed based on behaviorism and is used in management for changing behavior either by poking or giving rewards. Cox [3] proposed a model that is based on adult learning and human psychology. The study included the following eight learning theories relevant to coaching: andragogy, transformative learning, reflective practice, experimental learning, learning styles, life course development, values and motivation, and self-efficacy. The proposed model by Stober et al [4] focusses on a humanistic approach to the process of coaching with four guiding principles, including the nature of the coaching relationship, the client as a source and director of change, the client as a whole and distinct person, and the coach as the facilitator of the client’s growth. The model proposed by Knight [68] includes the method of instructional coaching. Visible learning (diagnosis, intervention, and evaluation) has been one of the research initiatives conducted in education in the past few decades. Simultaneously, instructional coaching (identify, learn, and improve) is becoming a popular form of professional development. Instructional coaching is used to support the realization of “visible learning” or other educational innovations. Standing [16] proposed a model to compare the use of “traditional” and “progressive” coaching styles to train a general male youth population to improve sprint and jump performances while assessing enjoyment in order to comment on the long-term application. The process includes the following steps: study design, participant selection, experimental procedure, data collection, statistical analysis, and performance measurement.

### RQ2: Which Conceptual Coaching Models Can Be Used to Explain the Coaching Process?

**Figure 2:**
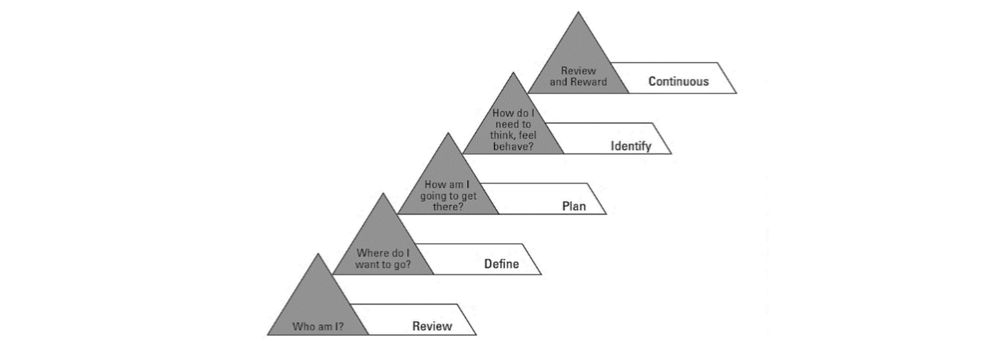
The five-level reviewing belief model by Whitten.

**Figure 3:**
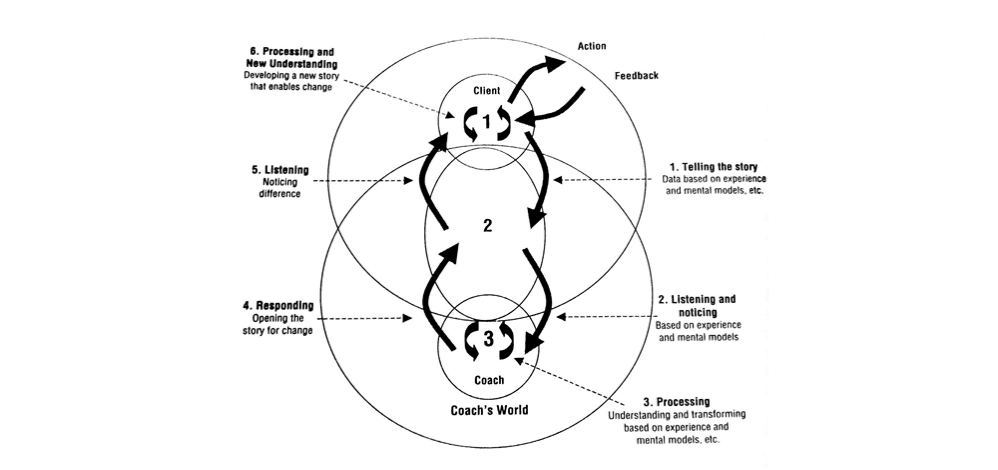
The dynamic coaching model by Cavanagh et al.

**Figure 4:**
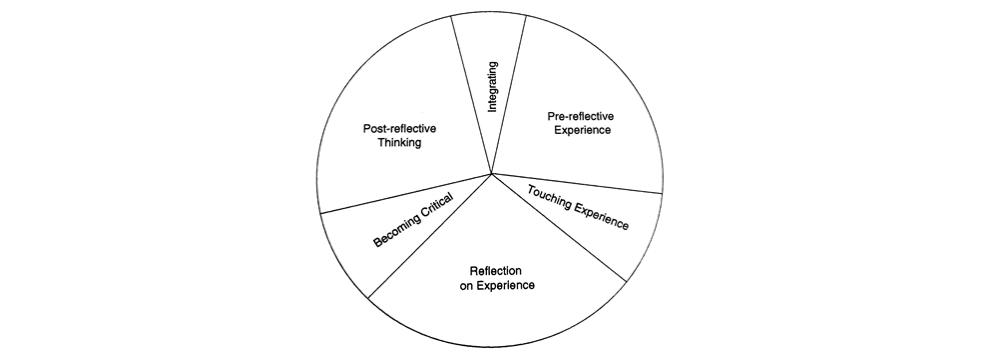
The experiential coaching cycle with six phases by Cox.

The actual definition of coaching concepts remains difficult to understand, and the working of the coaching interaction itself is still unknown [3]. The coaching approach may create a positive impact on the coaching environment and, subsequently, can improve the bottom-line performance of a target human group. An efficient coaching model is a tool to motivate personal learning, increase energy, improve ownership, and improve accountability. In contrast, no single coaching model can be labeled as the best, as coaching models change with the perspective and context of individual coaching. We found coaching model candidates for behavioral intervention that adequately explain the human coaching process. We divided our findings into the following two categories to have a better understanding of coaching process descriptive models: coaching process descriptive models and their application domain (Table 3 [3,7,8,71]; Figure 2 [8], Figure 3 [71], and Figure 4 [3]), and psychological approaches to describe coaching process models (Table 4 [4,66,67,72]).

**Table 3:** Coaching process descriptive models and their application domain.

Coaching process descriptive models	Application domain
*Five level reviewing belief model* [8]: The model explains the learned belief of a client who inherits or learns beliefs from his/her ancestors (parents) or teachers, or influential people to take any action from a decision. This model has the following five different levels from the bottom to the top: review (who am I), define (where do I want to go), plan (how am I going to get there), identify (how do I need to think, feel, behave), and continue (review and reward). When action is taken after a decision is made, the following five different levels are explored: beliefs and values, thoughts and expectations, emotions, behaviors, and actions. The model is shown in Figure 2. *ABCDE model* [8]: It explains how to use the tools and techniques of cognitive behavioral coaching to challenge negative thinking, make positive changes, achieve goals, and improve (ABCDE model: A, activating event or situation; B, the belief; C, the consequential emotion; D, disputing the belief; E, exchanging the thought). *Cognitive behavioral model* [7]: It utilizes a cognitive behavioral solution-focused model of coaching. It provides preliminary evidence that evidence-based life coaching can enhance mental health, quality of life, and goal attainment.	Cognitive behavioral coaching
*Dynamic coaching model* [71]: A coaching system is made up primarily of three spaces that contain three conversations that interact together to create the coaching conversation. The first reflective space is the internal conversation within the client. The second space is the shared space created in between the coach and client. The third space is the space within the coach. The complete coaching model is depicted in Figure 3.	Dynamic coaching
*Experiential coaching cycle* [3]: It has the following three noticeable constituent areas: prereflective experience, reflection on experience, and postreflective thinking, as depicted in Figure 4. The cycle additionally has the following three essential transition stages: touching experience, turning into critical, and integration. Transition phases regularly involve more emotional, cognitive, or physical effort than the constituent spaces and are particularly challenging for both coach and participant owing to the emotional struggle and inheritance of uncertainty.	Pragmatic coaching

**Table 4:** Psychological approaches for coaching process models.

Coaching process descriptive models	Psychological approaches
*Five elements model* [66]: The model explains the practice for human resource development, focusing on improving performance/examining results with a way of equipping human beings with the methods, knowledge, and possibilities they want to broaden themselves and become more effective. The unidirectional sequence of five elements includes establishing relationships, recognizing opening, observation or assessment, enrollment of clients or participants, and coaching conversations. *Goal focused executive coaching model* [4]: It explains how to improve personal or professional performance, personal satisfaction, and effectiveness in the client’s organization within a formally defined coaching agreement, and identify a set of goals using the following process: identification of an issue, setting of a goal, development of a cyclic action plan (act, monitor, evaluate, and change), and evaluation of the success score. *Organization response cycle* [67]: The model explains how to manage the pressure exerted on the department because of globalization, in order to produce faster, cheaper, and customized products and services. This model includes a cyclic loop of the following processes: learning (individual, team, organizational), innovation (products, and services), adaptation (responding to change and complexity), and results (enough or not).	Executive coaching
*Humanistic coaching model* [4]: The cyclic model of awareness-choice-execution (ACE) explains how to use the principles and tasks to teach participants how to harness their own growth process. In directing the process of coaching for change, the coach can ensure that the participant integrates “being (and awareness of that)” with “doing” such that the participant comes away with actual results.	Humanistic coaching
*Effective coaching model* [4]: It explains the core of the coaching process (“what is done!”) and represents how the contextual themes are legislated with the following seven key principles that strengthen the human coaching process: collaboration, accountability, awareness, responsibility, commitment, action, and results.	Contextual coaching
*Open innovation model* [72]: It explains several key factors for organization development throughout the following life cycle stages: birth (innovation, awareness, intuition, vision, commitment, risk, and flexibility), growth (decision making, delegation, team approach, state change, and ability to grow), maturity (feasibility, retain high performance, overcome obstacles, and responsiveness), revival (autonomy, integration, effective internal communication, and innovative high performance), and decline (renew strategy and structure, innovativeness, improve information processing, and increase tolerance level).	Organization development

### RQ3: What Are the Basic Coaching Methods to Make the Coaching Process Successful for the Promotion of a Behavioral Intervention?

A coach must sometimes strictly intervene with the client and insist on something or keep pressing on a point until a client is willing to look at it [2]. The process of human coaching includes an insight into how people learn and think, along with an understanding of what motivates them to achieve continuous high performance during behavioral intervention. Several coaching methods for the promotion of behavioral intervention are described in Table 5 [1-5,7,9,21-23,57,59,67,69,70,73]. The answer to “RQ3” contributes to “RQ4” and “RQ5” to analyze what limitations to overcome and what methods of offline behavioral intervention to include in eCoaching for the promotion of a healthy lifestyle.

**Table 5:** Coaching methods.

Method	Description
Systematic observation [7,23]	Systematic observation helps researchers to identify the instructional behaviors utilized by coaching practitioners within the practice environment. Observable and measurable data have the potential to solidify the scientific basis of the coaching process. Systematic observation must be capable of accurately and comprehensibly recording human behavior within a human coaching context.
Interpretive interview [2,3]	Achievement of the coaching process remains with observational data collection supplied with in-depth interviews that allow for the acquisition and interpretation of rich qualitative data based on the behavioral strategies of coaching.
Knowledge exchange [5]	In the search for an understanding of the coaching process, it is necessary to analyze and investigate the shared experience between the coach and participant.
Pragmatism [3]	Coaching is not a collection of techniques to apply or dogma to adhere to, rather it is a discipline that requires freshness, innovation, and relentless correction according to the outcomes being produced.
Understanding of human psychology [7,69,73]	Psychological principles on which coaching is based are essential. Without psychological understanding, coaches might go through the motions of coaching or use the behaviors associated with coaching, such as questioning, but fail to achieve the intended results.
Experience [5,7,67]	Experience is a skill that helps to improve competence and coaching outcomes, such as future advancement.
Trust [1]	Trust is one of the complex issues for coaches, whether internal and external. It teaches how not to use personal information and not to disclose it to illegitimate people.
Relationship [1]	The relationship must be based on mutual respect, trust, and mutual freedom of speech.
Expression [1]	Language impacts the goals of coaching by providing a means to assist the participant in being self-correcting and self-generating. It is important to provide new language to the participant for better understanding and learning.
Mentoring [3,5]	Mentoring is a more formal process, based on a one-to-one relationship with someone in the organization. While a mentor can use all the coaching types, their purpose is broader in scope than that of a coach.
Values and motivation [1]	Values are ideas about what is good and bad, and how things should be. Motivation is the internally generated feeling that stimulates participants to act. Motivation is related to the needs and values that have a correlation with intrinsic motivation.
Feedback [1,4]	Feedback is important for coaches to improve their learning environment.
Evidence based [4,7]	Evidence-based life coaching can enhance health, quality of life, and goal achievement.
Contextual [4,7,69]	Understanding the context is essential in coaching perspective, as it gives insights into why many participants either fail to use or resist the coaching approach.
Decision making [2,5,7]	Decision making includes data collection related to coaching, the privacy of the collected data, data cleaning, statistical analysis on the collected data, and the development of a machine learning model for prediction or regression analysis.
Goal based (goal setting) and evaluation [3,5,23,59]	Goals must be stated and measurable. Goals include clearly stated pathways to the preferred alternative by identifying strategies. Goal setting and goal evaluation are two essential parts of a behavioral intervention to determine the effectiveness of coaching. Goals must be specific, measurable, actionable, relevant, and time related. Evaluation of goals is important to understand the strengths and limitations of participants to set further attainable goals when necessary and reach the objectives.
Self-efficacy [9]	Self-efficacy has its core in social learning theory. It can be explained as the general or definite belief that people have concerning their capability to accomplish assigned tasks.
Personalization [21]	The concept of personalization or user tailoring is used in coaching to explain the variation in preferences between groups of participants and within the groups of participants to make recommendations more effective.
Persuasion [57]	Persuasion is a process that has been designed to change negative attitudes or behaviors of participants through advice, faith, and social influence. It is regularly used in the domain of public health where human-human or human-computer interaction is applied. It can be categorized as instruction style (authoritative and nonauthoritative), social feedback (cooperative and competitive), motivation type (extrinsic and intrinsic), and reinforcement type (negative and positive) [22].
Interaction and co-creation [70]	People are subject to self-regulation failures as follows: cravings, distractions, and deferring the right things. Therefore, people may need guidance through an eCoaching process to achieve the intended goal. Interaction is an integral part of pervasive computing that guides people to “do the right thing.” It requires improving automated logging of health (behavior) data and integrating this into coaching processes, as well as designing more intelligent and interactive coaching processes that incorporate user preferences and plans, contextual/situational priorities, and health data consequences. For successful design, the concept of co-creation or co-design is essential, where the system is designed together with its users.

### RQ4: How Can the Methods of Human Coaching Processes Be Incorporated Into eCoaching for Behavioral Intervention to Promote a Healthy Lifestyle?

The concept of eCoaching is constructed on the foundation of traditional coaching, and the technological revolution has boosted its performance and real-time acceptance. The World Health Organization (WHO) [49] claimed that chronic illnesses associated with modifiable lifestyle factors would be responsible for premature death worldwide. Therefore, change in negative health behavior should be given priority to avoid considerable losses caused by lifestyle diseases. An eCoach system can empower human beings to manipulate a healthy lifestyle with early health risk prediction and beneficial customized recommendations [22,61]. The pillars of eCoaching for behavioral intervention [56] are mostly inspired by the coaching methods as described in Table 5. They consist of data collection, data storage, analysis of data, goal setting, recommendation generation (intervention), monitoring, data privacy and ethics, goal evaluation, credibility, co-creation, feedback generation, and model evaluation [24,27,32,34,35,74]. Behavioral intervention is the process of intervening. As defined by WHO [49], a health intervention is an act performed for, with, or on behalf of a person or population, whose purpose is to assess, improve, maintain, promote, or modify health, functioning, or health conditions. Health interventions are used to promote a healthy lifestyle. Lifestyle or behavioral interventions include exercise, diet, and at least one other method (counseling, stress management, or healthy habits). Effective intervention planning is essential for an eCoach system for behavioral intervention to promote a healthy lifestyle change.

From the included “eCoach” articles, we found that the following methods are most appropriate for eCoaching processes: “personalization” (n=19) [21] “interaction and co-creation” (n=17) [70], “behavior change with technology” (n=17) [58], “goal setting” [59] and “evaluation” (n=16) [23,59], “persuasion” (n=15) [57], “automation” (n=14) [1], and “promotion of a healthy lifestyle” (n=14) [21]. These are relevant methods for eCoaching following a top-down ranking. “Personalized” recommendations are required to make intervention plans effective, and for that, personal “interaction” is necessary. For efficacy evaluation of eCoaching, personalized goal setting and goal evaluations are important. “Automation” is relevant to deliver automatic behavioral recommendations (“persuasion”) to participants for the promotion of a “healthy lifestyle.”

Methods in eCoaching, such as personalization, persuasion, goal setting and evaluation, interaction, and co-creation, are borrowed from traditional offline human coaching (Table 5). In eCoaching, persuasion is developed by trusting self-report or automation that observes human behavior using sensors, which is followed by health risk prediction with pattern recognition algorithms. The remaining four are core eCoaching methods. The first aspect is *automation* [1]. It helps to deliver automatic behavioral recommendations to users to maintain a healthy lifestyle. The decision support system (DSS) [22,61] within an eCoach system periodically monitors health and wellness parameters collected over time through sensors, questionnaires, and feedback forms, and predicts health risks. Once risk prediction is made, the DSS sends an automatic alert or recommendation to users. The second aspect is *behavior change with technology* [58]. Recent advancements in ICT have improved personal health care. The health care segment is still looking for an interactive, easy-to-use, optimized, cost-effective, and secure eCoach system for behavioral intervention for the promotion of a healthy lifestyle. The system should have the capability to normalize different formats of personalized data with appropriate ontological studies, ensuring the privacy of data. It should use AI algorithms based on ethical principles to analyze human psychology, monitor human behavior, and guide participants accordingly. Technology can support an eCoach by supporting coaching types, process management, human-computer interaction, remote collaborative work and communication, data collection and storage, data security and privacy, data analysis, recommendation generation, evaluation, and self-tracking. The third aspect is *promotion of a healthy lifestyle* [21]. Good health is the result of a healthy lifestyle, where caring about physical activities and nutrition are vital concerns. However, today, nutritional disorders are increasing rapidly. It is affecting children, adults, and older people, mainly due to limited nutrition knowledge and the lack of a healthy lifestyle. A commonly adopted approach for these imbalances is monitoring physical activity and daily habits, such as recording exercise and creating custom meal plans to count the number of macronutrients and micronutrients acquired in each meal. Behavioral interventions (nutritional and physical exercise coaching) through eCoaching have become popular (eg, Food4Living [17], TrainME [17], and RunningCoach [21]) for the promotion of a healthy lifestyle.

### RQ5: How Can eCoaching Promote a Healthy Lifestyle With Proven Coaching Methods Using ICT?

The point of interest of eCoach initiatives is to deliver high-quality, evidence-based, comfortable, cost-effective, and timely care to assist human beings in retaining a healthy way of life [1,23,57]. eCoaching methods represent an evolving trend as they diverge from the conventional methods that tend to devalue user behavior. Health eCoaching is a complex process that demands careful planning and cooperation of several scientific domains, such as psychology, computer science, ethics, and medical science [23]. An effective eCoach design focuses on co-creation, co-design, and personalization of the intervention by the user and the system [23,30,32,35,58]. There are six primary attributes when modeling an eCoach system as follows [24,27,32,34,35,58,59,74]: (1) identification of the target group of participants, (2) selection of the study case, (3) type of data to be collected and data collection method, (4) target of coaching, (5) approach of coaching, and (6) evaluation of the intervention plan.

According to the findings in studies on coaching regarding the importance of the personal relationship between a coach and trainee, personalization of coaching strategies, motivation, goal setting, and engagement of the eCoach with the trainee/coached citizen/patient has to be customizable and easily available. Therefore, the user interface design of an eCoach system must be unambiguous and easily understandable [23,24], and it must not include unwanted artifacts. It must be designed following a standard co-creation process. eCoaching systems are an emerging trend with a design criterion to reduce the involvement of human specialists with AI-inspired algorithms and robots for decision making based on supervised, unsupervised, and reinforced learning. In contrast, in several eCoach designs for behavioral intervention, human therapists or doctors, or other coaching experts are included [23,27,34,62]. The experts have access to the observation data, and they get involved or contribute to the coaching process.

eCoaching has other possibilities when compared to traditional coaching in terms of value addition, performance, and competence. Efficacy [52] study is a problem in both kinds of coaching to date, as revealed in the systematic literature review, for the following reasons: insufficient planning in study selection and study design [1,23], lack of conceptual/contextual clarity [24,25], inappropriate selection of sample size for statistical analysis [61], dearth of proper background education [74], lack of reliance and self-disclosure [1,22], absence of variation in a selected population [22,61], and lack of competence and experience with technology (digital illiteracy) [22,75].

## Discussion

### Overview

From the systematic literature review, we analyzed existing well-established traditional human coaching processes, descriptive models and the application domain, psychological approaches to describe coaching process models, methods in the coaching processes, and their applicability in eCoaching with the advancement of ICT to promote a healthy lifestyle. In this section, we discuss the findings associated with each individual research question.

### Discussion on RQ1

The answer to “RQ1” helped us to identify key methods in the coaching process, as defined in Table 6 [1-7,9,16,66-68]. We studied their significance in eCoaching. The identified key methods in the coaching process are based on a review of established coaching process description models relevant for this RQ. Identified coaching methods are used to answer “RQ4.” Appropriate coaching skills, knowledge to coach, method selection, proper implementation, personal interaction, and idea exchange are part of an effective coaching practice.

**Table 6:** Key methods associated with coaching.

Research group	Key methods associated with coaching
Potrac et al [66]	Behaviors, actions, and motivations
Cunningham et al [9]	Experience and racial diversity
Bartlett [1]	Trust, language, practice, and behavior
Côté [5]	Coach education and learning
Green et al [7]	Goal, psychology, evidence based, and cognition
Murphy [6]	Mentoring, evaluation, and leadership
Richards [67]	Intelligent coaching, learning, innovation, adaptation, Sustainability, and model performance
Flaherty [2]	Constraints of learning
Cox [3]	Pragmatism, experiencing, listening, clarifying, reflecting, and questioning
Stober et al [4]	Psychology, contextual, goal focus, cognition, and humanistic perspective
Knight [68]	Instructional coaching and visible learning
Standing et al [16]	Coaching, data collection, statistical analysis, and performance evaluation

### Discussion on RQ2

We depicted the top three coaching descriptive models from Table 3, such as the models of Cavanagh et al [71], Whitten [8], and Cox [3] on “dynamic coaching,” “cognitive-behavioral coaching,” and “pragmatic inquiry into the coaching process,” respectively. These models seemed to be suitable candidates to construct a personalized eCoaching process model for behavioral intervention for the promotion of a healthy lifestyle, contributing to the answer of “RQ4.”

### Discussion on RQ3

Systematic observation methods are recognized as useful research tools for providing quantitative descriptions of coaching behavior. Furthermore, coaching psychology mechanisms are also relevant for enhancing well-being, work performance, and personal life [7]. Therefore, researchers need to use systematic observation [64] and psychological coaching [7] to study coaching behavior in order to establish a database of meaningful coaching behaviors in different contexts. Besides the discussed strengths and potentials, constraints related to coaching as behavioral interventions are reviewed in the following text. *Language* is a medium of communication between people. Coaching may lead to language interpretability issues when selecting inappropriate language. Without proper communication, participants will be unable to perform the needed or desired tasks [2,66]. Regarding *understanding*, the humanistic nature of the coaching process remains little understood and an underresearched area [66]. Regarding *ethical dilemma*, researchers need to develop an ethical standard, adequate training, and presential coaching within a specific context. In many cases, coaches have not fully understood the performance-related psychological principles on which coaching is based [69]. Regarding *diversity*, the coaching study should consider diversity in all its forms, such as organizational and occupational tenure, age, race, educational background, attitude, and personality [9]. Regrading *human behavior*, many coaches do not ground their practice in behavioral science. Participants should be selected from a diverse community, as members of a single community cannot represent the general population [7]. Regarding *conceptual clarity*, besides the popularity, the human coaching process reveals lack of conceptual clarity, imprecise description, and paucity of efficacy studies [6]. There persists a wide gap between what practitioners believe coaching is and what many executives think about coaching [67]. Regarding *implementation challenge*, the most formidable challenge in the field of coaching is the challenge of translating research into practice. Thought needs to be given to the sharing of all visible learning aspects in a way that is manageable and a part of goal-directed learning [68]. Regarding *bias*, due to background and bias, experts do ignore psychological problems they do not understand and may worsen the intervention. Thus, psychotherapeutic intervention is essential [4]. Regarding *human psychology and pressure*, most coaching-related studies are inclined to psychology rather than the way to do coaching. Pressure-based coaching hampers team functioning by negatively influencing team loyalty through increased levels of tension within the group [48].

### Discussion on RQ4

**Figure 5:**
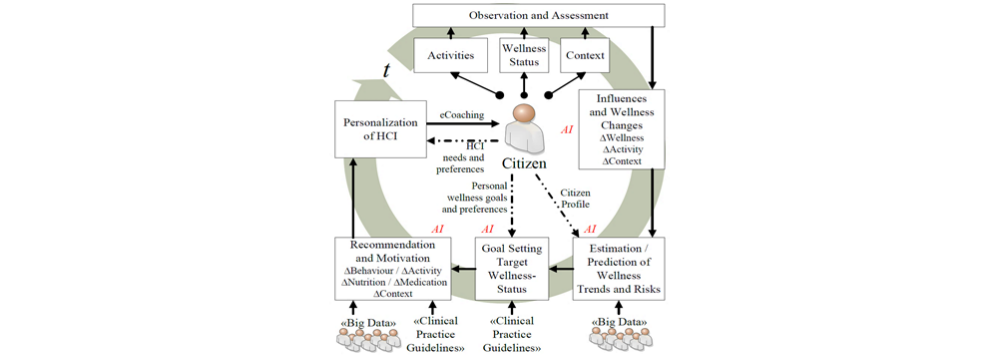
A holistic electronic coach (eCoach) model proposed by Gerdes et al. AI: artificial intelligence; HCI: human-computer interaction.

The answer to “RQ2” revealed that the coaching model could be implemented in the following two ways: (1) the coach at the center and participants (“citizens”) around, and (2) participants at the center and the coach around. Gerdes et al [61] proposed an eCoach concept based on monitoring, quantification of data, and AI, emphasizing human-centered design, with participants placed at the center, as depicted in Figure 5 [61]. The loop of the pictured eCoach model can be closed with an effective behavioral intervention plan, based on the selection of study cases, to guide people and deliver contextual and personalized recommendations to maintain a healthy lifestyle. This systematic literature review can help us understand how to solve the “What” (to coach) and “How” (to coach) questions related to eCoaching. In the eCoach model, as illustrated in Figure 5, the critical methods of coaching, as depicted in Figures 2-4, fit together for behavioral intervention.

### Discussion on RQ5

Digital methods [17] for behavioral intervention with personal coaching have emerged as effective and scalable options. They include methods for intensive behavioral counseling, supporting face-to-face consultations with accessibility, attractive and personalized interaction, efficient use of time, and managing costs. eCoaching has the potential to overcome problems, such as language, bias, conceptual clarity, ill-defined matters, freedom of expression, pressure, and tension, which are expected in traditional coaching, as discussed in the answer to “RQ3.” A smart eCoach may ideally deliver solutions asynchronously and on-demand with better flexibility and increased accessibility for personalized context-based coaching services. In this review, we have identified the following critical elements for effective eCoaching with AI [22,57,61,75-77]: real-time feedback, suggestion, and alert generation; preference sharing; comprehensible user experience (UX) design; interactive interaction (eg, intelligent chatbot); DSS; wellness vision (physical/social/emotional/spiritual); encouragement based on positive human psychology; assessment of human behavior based on physiological and contextual data; credibility; ethics; digital literacy to make the human-eCoach interaction effective; and generation of automatic, personalized, and context-specific recommendations to achieve health and wellness goals.

### Strengths and Limitations

This systematic literature review helps to identify key processes from coaching methods to solve existing challenges and use human coaching and eCoaching processes as behavioral interventions. Coaching consists of observation, offering hints, feedback, reminders, and new tasks and redirecting participant attention to a salient goal to enhance participant performance. Coaching is applied to unveil the potential of participants to maximize their performance. A coach facilitates experimental learning that results in future-oriented abilities. A coach can shape new visions and plans to generate desired results. Despite the underutilization of remote coaching of health and wellness in the health care system as an asset of clinical practice, the focus on coaching is justified. The integration of offline human coaching methods into the eCoaching process faces challenges related to privacy, ethics, coaching environment, skills, trust, motivation, intervention plans to change negative behaviors, human centeredness, and evaluation of preset goals. Despite the challenges, it is very promising to integrate human coaching methods into the eCoaching process [4,5,60]. An important limitation of this study is that we did not search the JMIR database, which has e-collections on the present topic. Future studies on this topic should search the JMIR database. This study serves as a basis for further research with a focus on designing an eCoach system based on the identified key coaching methods for the generation of personalized recommendations to achieve personal wellness goals.

### Conclusion

An ideal coach should have the potential to conceptualize and navigate through changing complex environments. The coaching process is adopted to bridge inadequacies in areas where human resource structures and practices should play a more active mediating role. The success of the coaching process is an art, and impact analysis is important to evaluate its accomplishment. An evaluation of the human coaching process is also necessary. Therefore, the learning environment of active coaches needs to be continuously revisited and adapted. Health monitoring and fitness coaching with AI has the potential to contribute to research in eHealth. An optimized system for health eCoaching and management of personal health data that ensures data protection and privacy are significant challenges associated with eCoach-related research. The prediction of human behavior by analyzing human psychology for the generation of useful lifestyle recommendations is another challenging task to overcome, as human behavior is continuously changing. This review will provide eHealth researchers with an overview of different coaching and eCoaching processes, with the aim to promote a healthy lifestyle. In addition, this review can be used as a basis for further research focusing on the design, development, testing, and evaluation of the performance of an eCoach in order to generate automatic, meaningful, evidence-based, contextual, and personalized recommendations to achieve personal health goals.

## Appendix

Multimedia Appendix 1 Search results from electronic databases.

## Abbreviations

AI: artificial intelligence
DSS: decision support system
eCoach: electronic coach
eCoaching: electronic coaching
ICT: information and communication technology
RQ: research question
WHO: World Health Organization
